# Focused ultrasound-aided immunomodulation in glioblastoma multiforme: a therapeutic concept

**DOI:** 10.1186/s40349-016-0046-y

**Published:** 2016-01-22

**Authors:** Or Cohen-Inbar, Zhiyuan Xu, Jason P. Sheehan

**Affiliations:** Department of Neurological Surgery, University of Virginia, Charlottesville, VA USA; Molecular Immunology & Tumor Immunotherapy Laboratory, Technion–Israel Institute of Technology, Haifa, Israel

**Keywords:** Focused ultrasound, FUS, HIFU, GBM, Immunomodulation, Synergistic immunotherapy

## Abstract

Patients with glioblastoma multiforme (GBM) exhibit a deficient anti-tumor immune response. Both arms of the immune system were shown to be hampered in GBM, namely the local cellular immunity mediated by the Th_1_ subset of helper T cells and the systemic humoral immunity mediated by the Th_2_ subset of helper T cells. Immunotherapy is rapidly becoming one of the pillars of anti-cancer therapy. GBM has not received similar clinical successes as of yet, which may be attributed to its relative inaccessibility (the blood-brain barrier (BBB)), its poor immunogenicity, few characterized cancer antigens, or any of the many other immune mechanisms known to be hampered. Focused ultrasound (FUS) is emerging as a promising treatment approach. The effects of FUS on the tissue are not merely thermal. Mounting evidence suggests that in addition to thermal ablation, FUS induces mechanical acoustic cavitation and immunomodulation plays a key role in boosting the host anti-tumor immune responses. We separately discuss the different pertinent immunosuppressive mechanisms harnessed by GBM and the immunomodulatory effects of FUS. The effect of FUS and microbubbles in disrupting the BBB and introducing antigens and drugs to the tumor milieu is discussed. The FUS-induced pro-inflammatory cytokines secretion and stress response, the FUS-induced change in the intra-tumoral immune-cells populations, the FUS-induced augmentation of dendritic cells activity, and the FUS-induced increased cytotoxic cells potency are all discussed. We next attempt at offering a conceptual synopsis of the synergistic treatment of GBM utilizing FUS and immunotherapy. In conclusion, it is increasingly apparent that no single treatment modality will triumph on GBM. The reviewed FUS-induced immunomodulation effects can be harnessed to current and developing immunotherapy approaches. Together, these may overcome GBM-induced immune-evasion and generate a clinically relevant anti-tumor immune response.

## Background

Glioblastoma multiforme (GBM) is the most common primary malignant brain tumor in adults. Despite standard of care treatment, the median survival of a patient harboring a GBM is less than 2 years. Although subdivided to different subtypes, these overall grim prognosis figures have stood fast and changed very little in the past few decades, proving resistant to most developments and revolutions incurred on modern medicine [1–3]. Current day first-line treatment for GBM patients includes a combination of surgery, chemotherapy, and radiotherapy [1–3]. Recent understanding of the role of epigenetic inheritance (e.g., promoter methylation) adds complexity to this issue. *O*^6^-methylguanine-DNA methyltransferase (MGMT) is a key repair enzyme that contributes to the resistance of tumors to alkylating agents (carmustine or temozolomide). MGMT promoter methylation silences the gene, thereby increasing the susceptibility of the tumor cells to these agents [1–3].

The unique nature of GBM and its inherent challenging features was evident as early as 80 years ago. In the 1930s, neurosurgeon Walter Dandy reported recurrence of contralateral GBM even after a hemispherectomy, emphasizing thus how infiltrative these tumors are. Patients with GBM exhibit a deficient anti-tumor immune response. Early reports of GBM patients with a post-operative surgical site infection who exhibit longer survival served as the first hint, sparking an interest in the topic. This phenomena was initially attributed to the non-specific immune recruitment invoked by the local bacterial lipopolysaccharide (LPS), targeting the tumor as well to some extent. Since these pivotal observations, a plethora of studies characterizing the immunosuppressive mechanisms in GBM have been conducted [3–19]. As discussed later, both arms of the immune system were shown to be hampered in GBM, namely the local cellular immunity mediated by the Th_1_ subset of helper T cells, and the systemic humoral immunity mediated by the Th_2_ subset of helper T cells [7, 8].

Immunotherapy is rapidly becoming one of the pillars of anti-cancer therapy. Its targeted nature and reduced treatment related toxicity, stemming from recruiting and activating own selective cytotoxic mechanisms, makes it intuitively an attractive option. GBM has not received similar clinical successes as of yet. This may be attributed to its relative inaccessibility (protected by the BBB), its poor immunogenicity, few characterized cancer antigens, or any of the many other immune mechanisms known to be hampered, briefly discussed next [3–19]. Preclinical studies suggest that immunotherapies can elicit significant anti-tumor responses in GBM, overcoming some of the barriers and tumor-related escape mechanisms, while others fail at specific points. Moreover, immunotherapies may have the potential to work synergistically with other treatment modalities such as high-intensity focused ultrasound (HIFU), surgery, radiotherapy, or alkylating agents.

Continuous-wave (CW) high-intensity focused ultrasound (HIFU) is emerging as a promising treatment approach. CW-HIFU is the only noninvasive thermal technique that allows for real-time imaging of the treatment progress using MR thermometry [20]. High-energy ultrasound beams are applied to focus acoustic energy on a well-defined region. Although, single ultrasound beams penetrate a tissue without causing any significant heat, focusing beams from multiple directions into a selected region results in a temperature rise (to >60 °C), and subsequently inducing coagulative necrosis [20–23]. Ultrasonic energy emitted from concave piezoelectric ceramics can be tightly focused with radial and axial dimensions of only 1–2 and 10–20 mm, respectively, based on the range of frequencies and transducer geometry. Complete tumor ablation is then achieved by employing multiple stereotactic sonications [20, 23–25].

The effects of FUS on the tissue are not merely thermal. Mounting evidence suggests that in addition to the thermal ablation mechanism, HIFU induces mechanical acoustic cavitation which has both mechanical and molecular implications and also boosts the host anti-tumor immune responses (Table 1) [26–47]. We will briefly review separately the different pertinent immunosuppressive mechanisms harnessed by GBM and the immunomodulatory effects of HIFU. Of note, many such HIFU-related mechanisms were shown for non-GBM tumors. We will next attempt at offering a conceptual synopsis of the synergistic treatment of GBM utilizing HIFU and immunotherapy.

**Table 1:**
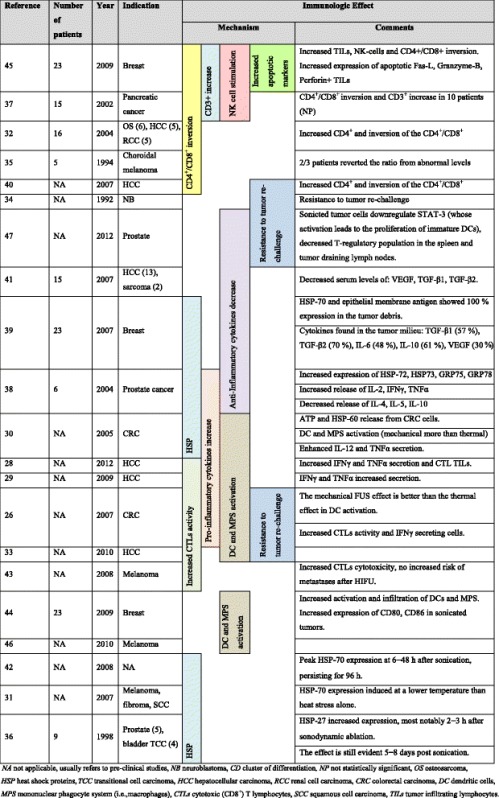
Focused ultrasound immunomodulatory effect—literature review

### Pertinent facts in immunology and brain-cancer immunotherapy

The initial interaction of the T cell receptor (TCR) with the major histocompatibility complex (MHC)-peptide complex is stabilized by the binding of CD4^+^ or CD8^+^ co-receptors molecules (termed as signal 1) [48]. This interactions are accompanied by the interaction of other receptors (termed as co-stimulatory or co-inhibitory signals), a process termed as signal 2. Efficient T cell activation requires as little as a single MHC-peptide complex for CD4^+^ T-helper cells and as little as 10 for cytotoxic T cells (CTLs) [49], yet a signal 1 interaction that lacks a stimulatory signal 2 will fail to elicit T cell activation and differentiation. Different co-stimulatory (e.g., CD28) and co-inhibitory (e.g., CTLA-4) signal 2 molecules directs differentiation to different avenues, ranging from a potent anti-tumor response (in CD28 activation), to anergy and even apoptosis (upon CTLA-4 or Fas activation) [48]. A local cytokine based pro-inflammatory or anti-inflammatory milieu plays a significant role in driving the immune response as well.

Mounting an effective brain anti-tumor immune response requires that certain requirements are met. As discussed later, the GBM cells developed mechanisms to evade or block every one of the following: first, the effector T cells, antibodies, or cytokines must penetrate the brain parenchyma before reaching the tumor bed. This entails crossing in active state through the BBB [50]. Some authors claim that the BBB is compromised/disrupted in the vicinity of the GBM [9] while others consider it a potent obstacle. Next, tumor-associated target antigens must be sufficiently different from self-antigens. Third, tumor cells must express sufficient MHC molecule to mount a specific CTLs effector-mediated response. Fourth, a local inflammatory response should then be instigated and properly regulated. Fifth, CTLs and T_H_1 cells must retain their anti-tumor effector function during migration through the brain parenchyma and its resident cells. The local cytokine microenvironment should support the T cell function. Finally, effector cell functionality must be retained during the encounter with the tumor cells, not suppressed actively or passively by the tumor cells.

### Immune mechanisms hampered in GBM

**Fig. 1:**
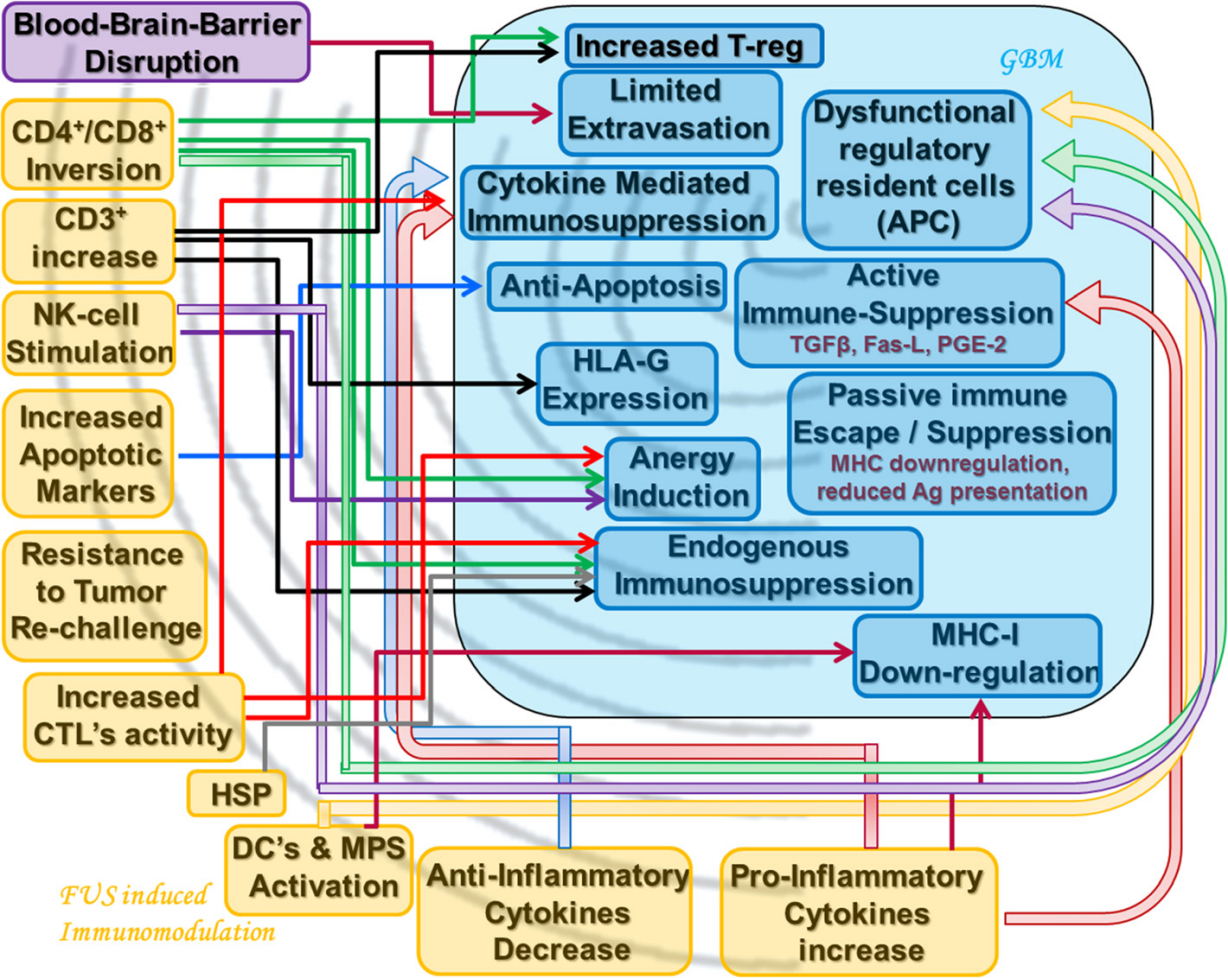
Synopsis. The key established mechanisms hampered in GBM and the key immune-modulating effects of HIFU are presented. A theoretical action-reaction scheme is presented, connecting certain known GBM-evasion mechanisms with the FUS-induced counter response. Note that a single FUS-induced effect may influence a multitude of immunosuppressive mechanisms and vice versa. Refer to text

A multitude of immunosuppressive mechanisms (both active and passive) as well as immune-evasion techniques are attributed to GBM cells (refer to Fig. 1) [3–19]. GBM patients were shown to exhibit overall low number of circulating T cells, rendering them vulnerable to any systemic challenge (infection, allergic reaction, or response to tumors) [10]. Fecci et al. [11] noted an abnormally high proportion (>2.5-fold increase) of circulating inhibitory T_reg_ cells (CD4^+^FoxP^+^) populations in GBM patients, unrelated to the co-administration of glucocorticoids [11]. The relative proportion of circulating T_reg_ cells is known to linearly correlate with in vitro suppression of effector T cell activation [12]. The most common genetic alteration in GBM (occurring in 80~95 % of tumors) is the loss of heterozygosity in chromosome #10 [7, 8, 13, 14]. Loss of the PTEN gene (phosphatase and tensin homologue) in this affected locus (10q23.3), serving as an inhibitor of the Phospho-inositol-3 kinase-signalling pathway may mediate a decrease in tumor cell immunogenicity by increasing the expression of B7-H1 (a co-inhibitory signal 2 molecule discussed previously), as well as increases anti-inflammatory (T_H_2 type) cytokines release [13, 14]. These mechanisms independently support the evolution of anergy and tolerance to the tumor.

Multiple genetic pathways, involved in cell-cycle regulation, growth-factor receptors presentation, and cytokine formation and regulation are known to be affected in GBM. Such pathways include the p16/pRb/CDK4 pathway, the p53/MDM2/p14ARF pathway, the epidermal growth factor receptor (EGF-R) gene [with unique variants like the EGFRvIII, responsive to Erlotinib], platelet-derived growth factor receptor (PDGF-R), and PI3-kinase/PTEN cascades [13, 14]. An immune-evasion mechanism suggested is the tumor’s ability to downregulate or express low levels of class-I MHC [15], hiding its existence from the cellular arm of the immune system, thus not detected by CD4^+^ T helper cells or CD8^+^ CTLs. This feature of immune evasion is compounded by the GBM ability to express aberrant non-classical MHC class I molecules (class Ib) termed as HLA-G. These decoy MHC molecules are structurally related to the classical MHC molecules (the class-Ia composed of HLA-A, HLA-B, HLA-C). HLA-G expression, renders tumor cells highly resistant to direct CTL-mediated alloreactive lysis, inhibits the alloproliferative response, and prevents efficient priming of cytotoxic T cells [16].

Additional GBM-related mechanisms involve the upregulation of anti-apoptotic proteins by the GBM tumor cells [17], rending the tumor cells immortal (e.g., unresponsive to death signals). Synergistically with all mentioned is the tumor-induced, cytokine driven, local immunosuppressive microenvironment. Reduced levels of pro-inflammatory cytokines (those driving to the T_H_1 differentiation) and increased local concentrations of anti-inflammatory cytokines and mediators (those driving to the T_H_2 differentiation) drive both the tissue and immune-elements that manage to reach the tumor site into an inactive or dysfunctional state [18]. Cytokines secreted, be it directly or indirectly by the GBM tumor cells, mediate immune-anergy and tumor proliferation [10, 19]. Of note, the complex interplay between the different mechanisms stated is complex and largely unknown.

### FUS-induced anti-tumor effects

Focused ultrasound exerts an anti-tumor effect utilizing three distinct complementary “modes” of action: thermal ablation, acoustic cavitation, and immunomodulation. Thermal ablation, the most obvious and characterized mode, is similar to many other lesional technologies in that the tissue at the focal point is targeted with the goal of causing coagulative necrosis. The second mechanism, acoustic cavitation, is in essence mechanical lysis of the tissue caused by harnessing acoustic cavitation, namely the rapid expansion and contraction of microbubbles/gaseous nuclei in cells through acoustic pressure within the targeted tissue [51]. This process enhances the local heating [52] as well as leads to collapse of the intracellular machinery (i.e., mitochondria, endoplasmic reticulum, as well as nuclear and cell membranes) [20]. Harnessing the advantages of mechanical cavitation, the FUS aided disruption of the BBB (at lower exposure) serves as another synergistic mechanism to prime an anti-GBM response (discussed next) [53, 54]. In addition to disrupting the BBB, these micro-bubbles can potentially be loaded with tumor antigens, allowing for immune-homing, mounting a more focused, effective immune response [53–55]. The third mechanism employs sub-lethal mild hyperthermia, i.e., a uniform low-level heating of a region of interest [39, 56, 57]. Pulsed-mode HIFU with negative pressures equal or higher than that used for thermal ablation (7–12 MPa) was shown to boost the systemic anti-tumor immune response through multiple mechanisms (Table 1) [26–47]. Immunologically speaking, although considered different, each of these modes shares a final common pathway in terms of initiating an immune response. This response is instigated through the release of tumor debris rich in unique antigens and danger signals into the inter-cellular matrix [51].

### FUS-induced immuno-modulating effects

Many preclinical and clinical studies have demonstrated that FUS can facilitate and amplify an immune anti-tumor response, prolonged overall survival and protection from growth of new tumors when re-challenged (Table 1) [26–47]. One should note that all immune-modulating effects discussed hereafter were described on other tumor types. The assumption that these effects are FUS/HIFU based and are tumor type independent, was not validated objectively. All three HIFU-related modes of action can be assigned an immune-modulating mechanism. The HIFU-induced thermal ablation causes surviving tumor cells to upregulate danger signals such as heat shock proteins (HSP) and adenosine triphosphate (ATP). HSPs have long been viewed as potent innate immunity tools, serving to increase the tumor immunogenicity [30, 31, 58]. Second, mechanical cavitation allows for better BBB penetration for antigens and immune cells as well as results in lysis-related tumor debris. These debris serve then as potent antigens for the immune system, activating dendritic cells (DCs) [29]. Third, FUS mitigates tumor-induced immunosuppression. We will now briefly discuss each of the last two proposed mechanisms. A thorough discussion of the direct ablative thermal effect is not in the scope of this review. Table 1 presents a brief overview of key preclinical and clinical studies, per different tumor type, segregated based on the proposed FUS-induced immunomodulatory effect. Of note, this table is not restricted to studies conducted in GBM or in the CNS, rather serves as a proof of concept for FUS-based immune-triggered events.

#### Microbubbles and BBB disruption

The addition of microbubbles (MB) to FUS can help generate more local heating in the area of focus [52], but at lower exposures, FUS with MB can be used solely for BBB disruption. These microbubbles, delivered intravenously, are composed of lipid-encased perfluorocarbon gas, and measure approximately 1–5 μm in diameter [59]. Recent reports suggest that the permeability of the blood vessels can be substantially increased by FUS in the presence of MB. Even at greatly reduced acoustic pressures, barely above the pressure thresholds employed in diagnostic ultrasound, these MBs can be used to enhance targeted delivery of chemotherapeutic agents [60–63]. FUS exposure bursts of 10 ms, repeated at a frequency of 1 Hz and used for 20–30 s durations are the typical settings used. FUS with MB impose mechanical forces on the vascular endothelium which then results in a transient opening of the inter-endothelial tight junctions [64]. This translates to a local and reversible BBB disruption. The size and resonance frequency of the microbubbles can be altered, allowing for agents up to 2000 kDa to enter, in order to facilitate different goals [65]. Larger microbubbles require less acoustic pressure to achieve BBB opening. The FUS and MB BBB-disruption effects last for several hours and can be localized to the tumor region, prior to returning to the pre-FUS state [66]. Dynamic contrast-enhanced MRI has been shown to be able to monitor the kinetics of BBB disruption [67].

Several preclinical studies have demonstrated the feasibility of BBB disruption by FUS and MB for the administration of chemotherapeutics in the treatment of glioma. Aryal et al. [68] demonstrated increased survival in a rat glioma model, upon treatment with a combination of FUS and liposomal doxorubicin. Such liposomal doxorubicin would not cross the BBB without FUS-mediated disruption. Liu et al. [69] reported an increased concentration of temozolomide in a glioma model after FUS, which correlated with a diminished tumor progression and increased animal survival [69]. Yang et al. [70] reported the successful use of HIFU in delivering interleukin-4 (IL-4) receptor-targeted liposomal doxorubicin for enhanced targeted drug delivery and anti-tumor effect in GBM mouse model [70]. FUS with MB method of BBB disruption was shown to be applicable in delivering nanoparticles, DNA, plasmid vectors, and antibodies [71, 72].

Chen et al. [73] reported their successful preclinical feasibility study of FUS-induced BBB disruption in order to enhance IL-12 delivery in a C-6 glioma rat model. FUS-induced BBB opening had no obvious effect on the T lymphocytes population in normal animals, either in the brain or systemically. Yet, it triggered mild changes in the tumor-infiltrating lymphocyte (TIL) population, particularly in numbers of CD3^+^CD8^+^ CTLs in the tumor region. IL-12 administration triggered a profound increase in all TIL populations, including CD3^+^CD4^+^ T helper cells, CTLs, and CD4^+^CD25^+^ T_reg_. The combined FUS-BBB opening with IL-12 administration produced the most significant IL-12 increase, CTL increase and CTL/T_reg_ ratio increase, thus contributing to the most significant suppression of tumor progression and increased animal survival. These reports provided evidence that FUS-mediated BBB opening can enhance immune-modulating agent delivery to the brain.

#### Cytokines and stress response

HSPs are intracellular molecular chaperones, able to bind tumor peptide antigens and enhance tumor cell immunogenicity [74–79]. Antigen-presenting cells (APCs; namely DCs, macrophages and CD4^+^ T helper cells) endocytose the HSP-tumor peptide complex and present the chaperoned peptides directly to tumor-specific CTLs. This evokes potent cellular immune responses against tumor cells [74–79]. Autologous HSP-peptide complexes generated from a single individual tumor were shown to generate a therapeutic immune response in animal models [39]. Because random mutations in GBM cells usually produce patient-unique tumor associated antigens, HSP vaccination may be a rationally personalized approach that may obviate the requirement to identify the unique antigens [74–79].

HIFU was shown to upregulate the expression of HSP70 both in vitro and ex vitro [30, 31, 42]. An increased HSP-70 expression was detected on the surviving cell membrane of 23 patients with breast cancer treated with HIFU ablation. HSP expression was mainly found in the central necrosis zone, with only sparse positively stained cells observed in the periphery [39]. The most striking change noted was the positive expression of EMA and HSP-70 on the treated cancer cells in all 23 patients after HIFU ablation [39]. EMA is a known mucinous glycoprotein considered as a differentiation tumor marker and a histological prognostic agent.

#### Peripheral and intra-tumoral immune cell populations

FUS was shown to enrich the TILs population in immune-potent pro-inflammatory potent anti-tumor effector cells. In human breast cancer specimens collected 1–2 weeks after FUS treatment [45], a significant increase in TILs of both T and B subsets at the margin of the ablated region was shown, as compared to FUS-untreated tumor samples. Immunohistochemistry analysis showed that a subset of these cells was activated CTLs (CD57^+^), expressing perforin and granzyme B molecules, indicative of a cytotoxic effector function [45]. A randomized study of 48 patients comparing those who underwent FUS treatment prior to radical mastectomy to patients who underwent surgery alone showed that those who had received FUS treatment prior to surgery had a significantly higher level of TILs of T, B, and NK cells subsets [80].

This TILs enrichment phenomenon is not limited to breast cancer. It was shown in patients treated with FUS suffering posterior uveal melanoma [35], pancreatic carcinoma [37], osteosarcoma [32], hepatocellular carcinomas (HCC) [32], and RCC [32]. Some combination of increased percentages of CD3^+^ T cells, CD4^+^ T cells, a higher CD4^+^/CD8^+^ ratio, or NK cell stimulation [37] were observed [32, 35]. Refer to Table 1 for review of landmark studies and related mechanisms.

#### Augmentation of dendritic cell activity

FUS was shown to enhance the infiltration capabilities of dendritic cells (DCs) [15, 18] as well as other antigen presenting cells [44] in the treated tumor. Enhanced infiltration was followed subsequently with DCs migration to the draining lymph nodes, presenting tumor-associated antigens to a wide variety of circulating T cells [26, 43]. Zhang et al. [33] demonstrated that tumor debris induced by HIFU could serve as an effective immunogenic vaccine. This vaccine was shown to elicit tumor-specific immune responses, including induction of CTL cytotoxic activity, DCs-enhanced activation, and to protect naïve mice against a lethal tumor challenge. When tumor debris were loaded with immature DCs, this vaccine could induce maturation of DCs to a significant extent as well as increased CTLs cytotoxicity (manifested by elevated TNF-α and IFN-γ secretion) [29]. The authors noted that while this vaccine was able to initiate a host specific immune response after H22 challenge in the vaccinated mice which resulted in tumor growth rate reduced, no survival advantage could be observed [29]. Thus, HIFU induces activation and stimulation of various APCs, leading to an increased expression of costimulatory molecules and enhanced secretion of IL-12 (via DCs) and TNF-α (macrophages) [30]. The potency of dendritic cell infiltration and activation was shown to be further improved when sparse-scan mode FUS was used compared to dense-scan mode [46, 53], yet a detailed technical and physical discussion of FUS modes is beyond the scope of this review.

#### Resistances to tumor re-challenge and increased CTLs potency

A portion of the immunomodulatory effects of FUS noted in different reports are lacking exact molecular mechanisms as of yet, collectively termed as resistance to tumor re-challenge. Some overlap with CTLs potentiation is suspected and hence these mechanisms are discussed together. Zhang et al. [33] reported their results with mice implanted with an HIFU-treated H22 hepatocellular carcinoma (HCC) tumor cells. The animals received a subcutaneous tumor challenge 10 days after vaccination. Tumor growth was significantly delayed in mice vaccinated with previously HIFU-treated tumor cells. Mice that were treated with HIFU showed an 88 % survival rate at 60 days as compared to 36 and 16 % for the sham-HIFU and control groups, respectively [33, 46]. Survival, however, was not different between the groups [33]. Other mouse models concurred with this observation and have shown a significant decrease in the growth rate of tumors during a second challenge [34]. Yang et al. [34] reported the use HIFU to treat C1300 neuroblastoma cells implanted in mouse flanks, followed by the re-challenge of the same tumor cells. The authors noted a significantly slower growth of re-implanted HIFU-treated tumors compared with the controls.

Several reports of potentiated CTLs effector activity and potency after HIFU support this mechanism (Table 1). Increased effector function can be measured by increased IFNγ and TNFα secretion [26, 28, 29] or increased direct CTLs-mediated cytotoxicity [43]. Xia et al. [28] reported that the cytotoxicity of CTLs and the number of activated tumor-specific CTLs was significantly increased in the H22 tumor bearing mice treated with HIFU. Adoptive transfer of the activated lymphocytes was shown to provide better long-term survival and lower metastatic rates in the mice re-challenged by the same tumor cells as compared with sham-HIFU and control groups, due to the induced anti-tumor cellular immunity in the mice [28]. Similar results were reported in mice implanted MC-38 colorectal adenocarcinoma (CRC) and melanoma after HIFU ablation. HIFU treatment was shown to induce an enhanced CTLs activity in vivo, providing protection against subsequent tumor re-challenge [26, 43].

### Synopsis and future directions

In an attempt to direct hypothesis-based approach to FUS-induced immunomodulation in GBM, the key established mechanisms hampered in GBM and the key immune-modulating effects of FUS discussed in this review are presented in Fig. 1. A theoretical action-reaction scheme is presented; connecting certain known GBM-evasion mechanisms with the FUS-induced counter response. A single FUS-induced effect may influence a multitude of immunosuppressive mechanisms and vice versa. This scheme is given in an attempt to present a theoretical basis for the effectiveness of HIFU immunomodulation and synergistically supporting immunotherapies overcoming many of the GBM mediated immune-resistance mechanisms.

Future research still needs to be done to both dissect the different FUS-induced molecular and immunological mechanisms at play. Further research is needed to optimize the FUS treatment method, find other combinational therapies, defining how to most effectively use it in combination with immunotherapy [51].

## Conclusions

No single treatment modality will cure GBM. It is increasingly apparent that surgery alone, HIFU ablation, chemotherapy, or immunotherapy will not solely triumph. We reviewed the FUS-induced immunomodulation effect, which can be harnessed to the current and developing immunotherapies approaches. Together, these modalities may overcome GBM-induced immune-evasion and generate a clinically relevant anti-tumor immune response. Further study to the synergistic collaboration of different therapeutic approaches and the elaborated molecular immune interplay will shed light on this formidable challenge.

## Abbreviations

APCs: antigen-presenting cells
ATP: Adenosine triphosphate
BBB: blood-brain barrier
CD: cluster of differentiation
CRC: colorectal adenocarcinoma
CTLA4: cytotoxic T-lymphocyte-associated protein 4
CTLs: cytotoxic T cells
CW: continuous-wave
DCs: dendritic cells
EGFR: epidermal growth factor receptor
FUS: focused ultrasound
GBM: glioblastoma multiforme
HCC: hepatocellular carcinomas
HIFU: high intensity focused ultrasound
HLA: human leukocyte antigen
HSP: heat shock proteins
IFN: interferon
IL: interleukin
LPS: lipopolysaccharide
MB: microbubbles
MGMT: *O*^6^-methylguanine-DNA methyltransferase
MHC: major histocompatibility complex
PTEN: phosphatase tensin
RCC: renal cell carcinoma
TCR: T cell receptor
T_H_: T helper cell
TILs: tumor-infiltrating lymphocytes
TNF: tumor necrosis factor
